# p38 and Casein Kinase 2 Mediate Ribonuclease 1 Repression in Inflamed Human Endothelial Cells via Promoter Remodeling Through Nucleosome Remodeling and Deacetylase Complex

**DOI:** 10.3389/fcell.2020.563604

**Published:** 2020-10-15

**Authors:** Katrin Bedenbender, Isabell Beinborn, Evelyn Vollmeister, Bernd Schmeck

**Affiliations:** ^1^Institute for Lung Research, Universities of Giessen and Marburg Lung Center, Philipps-University Marburg, Marburg, Germany; ^2^Department of Pulmonary and Critical Care Medicine, Department of Medicine, University Medical Center Giessen and Marburg, Philipps-University Marburg, Marburg, Germany; ^3^Member of the German Center for Lung Research, Member of the German Center for Infectious Disease Research, Marburg, Germany; ^4^Center for Synthetic Microbiology, Philipps-University Marburg, Marburg, Germany

**Keywords:** ribonuclease 1, endothelium, inflammation, p38 kinase, casein kinase 2, nucleosome remodeling and deacetylase complex, histone deacetylation, CHD4

## Abstract

Vascular pathologies, such as thrombosis or atherosclerosis, are leading causes of death worldwide and are strongly associated with the dysfunction of vascular endothelial cells. In this context, the extracellular endonuclease Ribonuclease 1 (RNase1) acts as an essential protective factor in regulation and maintenance of vascular homeostasis. However, long-term inflammation causes strong repression of RNase1 expression, thereby promoting endothelial cell dysfunction. This inflammation-mediated downregulation of RNase1 in human endothelial cells is facilitated via histone deacetylase (HDAC) 2, although the underlying molecular mechanisms are still unknown. Here, we report that inhibition of c-Jun N-terminal kinase by small chemical compounds in primary human endothelial cells decreased physiological RNase1 mRNA abundance, while p38 kinase inhibition restored repressed RNase1 expression upon proinflammatory stimulation with tumor necrosis factor alpha (TNF-α) and poly I:C. Moreover, blocking of the p38 kinase- and HDAC2-associated kinase casein kinase 2 (CK2) by inhibitor as well as small interfering RNA (siRNA)-knockdown restored RNase1 expression upon inflammation of human endothelial cells. Further downstream, siRNA-knockdown of chromodomain helicase DNA binding protein (CHD) 3 and 4 of the nucleosome remodeling and deacetylase (NuRD) complex restored RNase1 repression in TNF-α treated endothelial cells implicating its role in the HDAC2-containing repressor complex involved in RNase1 repression. Finally, chromatin immunoprecipitation in primary human endothelial cells confirmed recruitment of the CHD4-containing NuRD complex and subsequent promoter remodeling via histone deacetylation at the *RNASE1* promoter in a p38-dependent manner upon human endothelial cell inflammation. Altogether, our results suggest that endothelial RNase1 repression in chronic vascular inflammation is regulated by a p38 kinase-, CK2-, and NuRD complex-dependent pathway resulting in complex recruitment to the *RNASE1* promoter and subsequent promoter remodeling.

## Introduction

Endothelial cells (ECs) significantly participate in regulation and control of vascular homeostasis and are rapidly activated upon inflammation to support the immune system. Thereby, ECs promote the inflammatory response via interaction with circulating leukocytes that infiltrate into the underlying tissue to secrete high amounts of proinflammatory compounds (Pober and Sessa, 2007). Despite the necessity of these processes to ensure a sufficient immune response, prolonged inflammation may also destruct the homeostatic functions of the endothelium. This further promotes progression of vascular diseases, like thrombosis or atherosclerosis (Poredos, 2002; Sitia et al., 2010; Zernecke and Preissner, 2016). In context of vascular inflammation, the endothelial extracellular RNA (eRNA)–Ribonuclease 1 (RNase1) system is known as a major key player to induce the immune response and likewise protect the EC layer (Zernecke and Preissner, 2016). Upon acute inflammation, ECs release eRNA as danger signal to initiate the immune response at the site of vascular injury. Simultaneously, vessel-protective RNase1 is released by vascular ECs to protect the endothelium from an overwhelming inflammatory response via degradation of eRNA (Landre et al., 2002; Cabrera-Fuentes et al., 2014; Gansler et al., 2014). However, upon long-term inflammation, accumulating eRNA enhances immune cell recruitment to the vascular wall and secretion of proinflammatory cytokines like tumor necrosis factor (TNF)-α or interleukin (IL)-1β. These cytokines further act on the EC layer via recruitment of histone deacetylase (HDAC) 2 to the *RNASE1* promoter, resulting in massive RNase1 repression (Gansler et al., 2014; Bedenbender et al., 2019). Here, HDAC2 specifically reduces acetylation (ac) of the transcriptional activation markers histone 4 (H4) and histone 3 lysine 27 (H3K27) to induce chromatin remodeling and subsequent gene repression (Kouzarides, 2007; Wang et al., 2008; Bedenbender et al., 2019). However, the precise molecular mechanism by which HDAC2 is recruited to the *RNASE1* promoter in inflamed human ECs remains unknown. This study aims to investigate the underlying signaling cascade involved in RNase1 regulation via analysis of responsible signaling pathway(s), modulators of HDAC2 activity, and chromatin remodeling complexes upon EC inflammation.

Inflammation-mediated RNase1 repression is assumed to be a specific inflammatory reaction in human ECs, mediated via stimulation with the proinflammatory cytokines TNF-α and IL-1β, as well as the toll-like receptor 3 ligand polyinosinic polycytidylic acid (poly I:C) (Gansler et al., 2014; Bedenbender et al., 2019). Thus, RNase1 regulation by common signaling pathway(s) activated by the afore-mentioned stimuli is suggested. TNF-α, IL-1β, and poly I:C are potent regulators of gene expression, tightly associated to inflammation-mediated cellular responses. Thereby, they conduct their functions primarily via two distinct signaling cascades through nuclear factor kappa B (NF-κB) or mitogen-activated protein kinase (MAPK) signaling, such as c-Jun N-terminal kinase (JNK) or p38 MAPK (Kawai and Akira, 2006; Weber et al., 2010; Brenner et al., 2015). Gansler et al. (2014) already demonstrated an NF-κB-independent signaling mechanism for inflammation-mediated RNase1 repression, supporting the hypothesis of a MAPK-dependent RNase1 regulation.

Apart from signaling cascade(s), it is still unclear how HDAC2 is recruited to the *RNASE1* promoter. Enzymatic activity of class I HDACs, including HDAC2, is regulated through three different stages, subcellular localization, association with multiprotein complexes and post-translational modifications, e.g., serine phosphorylation (Segre and Chiocca, 2011). Especially, phosphorylation of HDAC2 is associated with its enzymatic activity and conducted by the highly conserved, constitutively active serine/threonine kinase casein kinase 2 (CK2) (Tsai and Seto, 2002; Litchfield, 2003; Brandl et al., 2009). Thereby, CK2 comprises the ability to regulate several hundred target proteins and is therewith involved in diverse cellular processes, including inflammation (Litchfield, 2003; Singh and Ramji, 2008). Additionally, CK2-mediated HDAC2 phosphorylation is also described to support its association with several multiprotein complexes (Segre and Chiocca, 2011). A prerequisite for sufficient enzymatic function and recruitment to the chromatin, as HDACs do not comprise any DNA-binding ability (Sengupta and Seto, 2004). The most abundant HDAC2-associated repressor complexes are the REST co-repressor (CoREST) complex, the SIN3 complex and the nucleosome remodeling and deacetylase (NuRD) complex, which in part also coexist together with CK2 (Zhang et al., 1997; Tong et al., 1998; You et al., 2001; Sengupta and Seto, 2004; Sun et al., 2007; Yang and Seto, 2008).

In this study, we investigated the underlying signaling cascade of inflammation-mediated RNase1 regulation in human ECs: First, we addressed the impact of common signaling pathways involved in RNase1 regulation. Second, we investigated how HDAC2 can be regulated during EC inflammation, and third, which co-repressor complex is involved in chromatin remodeling of the *RNASE1* promoter. Our findings provide evidence that RNase1 expression is mediated via JNK signaling under physiological conditions, while its reduction during prolonged EC inflammation is regulated via p38 MAPK. We further provide evidence that the HDAC2-regulating kinase CK2, as well as the NuRD co-repressor complex acted as crucial regulators of inflammation-mediated RNase1 repression, on mRNA as well as chromatin level, respectively. Thereby, the NuRD complex component chromodomain helicase DNA binding protein (CHD) 4 accumulates at the *RNASE1* promoter along with reduction of H4ac and H3K27ac in a p38-dependent manner. These findings indicate a complex regulatory network involved in endothelial RNase1 regulation.

## Materials and Methods

### Ethics Statement

All umbilical cords were donated from healthy individuals who were fully informed and consented to donation. Donated tissue was handled in accordance with the local ethics regulations of the Philipps-University Marburg (permit number: AZ 20/16).

### Cell Culture

Cells used in this study were cultivated in a humidified incubator at 37°C with 5% CO_2_. Human umbilical vein endothelial cells (HUVEC) were isolated and cultured in EC growth medium from PromoCell (Heidelberg, BW, Germany) supplemented with 1% penicillin and streptomycin (Thermo Fisher Scientific, Waltham, MA, United States) as described previously (Bedenbender et al., 2019) and cultured up to passage 4 for all indicated experiments. For stimulation experiments 3.8 × 10^4^ cells/cm^2^ were seeded overnight. Cells were stimulated with human recombinant TNF-α [10 ng/ml] (R&D Systems, Inc., Minneapolis, MN, United States) or poly I:C [10 μg/ml] (InvivoGen, San Diego, CA, United States) as indicated. For inhibitor assays, HUVEC were pretreated for 1 h with the NF-κB inhibitor BAY11-7082 [1 μM, 5 μM], the JNK inhibitor JNK inhibitor II [10 μM, 30 μM], the p38 inhibitor SB202190 [10 μM, 20 μM] (Merck KGaA, Sigma Aldrich, Darmstadt, HE, Germany) prior to indicated stimulation for 24 h. Dimethyl sulfoxide (DMSO) (Carl Roth GmbH & Co., KG, Karlsruhe, BW, Germany) was used as solvent control. For chromatin immunoprecipitation (ChIP) assays, HUVEC were stimulated with 10 ng/ml TNF-α (R&D Systems, Inc.) for 10 min. For p38 inhibitor ChIP assays, HUVEC were treated for 2 h with 20 μM p38 inhibitor SB202190 (Sigma Aldrich) or DMSO (Carl Roth GmbH & Co., KG) as solvent control prior to 10 ng/ml TNF-α treatment (R&D Systems, Inc.) for 10 min.

The hybrid EC line EA.hy926 (American Type Culture Collection (ATCC), Manassas, VA, United States) was cultured in Dulbecco’s Modified Eagle’s Medium with high glucose (DMEM) supplemented with 10% fetal calf serum (Gibco^TM^, Thermo Fisher Scientific) for stimulation experiments. Cells were cultured up to passage 25 for all indicated experiments. Cells were seeded with 3.8 × 10^4^ cells/cm^2^ overnight followed by indicated treatments: For casein kinase 2 (CK2) inhibitor assays, EA.hy926 were pretreated for 30 min with 10 μM CK2 inhibitor TBB (4,5,6,7-Tetrabromobenzotriazole, Merck KGaA) or DMSO (Carl Roth GmbH & Co., KG) as solvent control prior to 24 h TNF-α [10 ng/ml] (R&D Systems, Inc.) or poly I:C [10 μg/ml] (InvivoGen) stimulation.

### siRNA Knockdown

EA.hy926 (3.8 × 10^4^ cells/cm^2^) were seeded overnight and transfected for 24 h with ON-TARGETplus small interfering (si) RNA SMARTpools (Dharmacon^TM^, Horizon Discovery Group Company, Lafayette, CO, United States) against CK2 alpha subunit 1 (CSNK2A1), CK2 alpha subunit 2 (CSNK2A2), CK2 beta subunit (CSNK2B), REST co-repressor 1 (RCOR1), SIN3 transcription regulator family member (SIN3) A, SIN3B, CHD3, CHD4 or an ON-TARGETplus Non-targeting Control Pool (siCTRL) as transfection control (50 pmol for single- or 25 pmol for double-transfection) using Lipofectamine^TM^ RNAiMAX (Invitrogen^TM^, Thermo Fisher Scientific) in Opti-MEM^TM^ I reduced serum medium and DMEM with 10% fetal calf serum (Gibco^TM^, Thermo Fisher Scientific). After transfection, fresh medium was added, and cells were stimulated for additional 24 h with 10 ng/ml TNF-α (R&D Systems, Inc.) or left untreated as control.

### RNA-Isolation and Quantitative Reverse Transcription PCR

Total RNA was isolated from HUVEC or EA.hy926 and cDNA was generated as described previously (Bedenbender et al., 2019). Transcript expression of RNase1, IL-8, cyclo-oxygenase 2 (COX-2), CSNK2A1, CSNK2A2, CSNK2B, RCOR1, SIN3A, SIN3B, CHD3, CHD4, and RPS18 as internal control was analyzed by quantitative reverse transcription PCR (qRT-PCR) with respective primer pairs (Table 1, metabion international AG Planegg/Steinkirchen, BY, Germany), using LUNA^®^ Universal quantitative PCR (qPCR) Master Mix (New England Biolabs, Ipswich, MA, United States) and the QuantStudio^TM^ System and QuantStudio^TM^ Design and Analysis Software v1.3.1 (Thermo Fisher Scientific) according to manufacturer’s instructions. The fold-induction was calculated using the 2^–ΔΔct^ method and qRT-PCR results were normalized to the corresponding control cells (Livak and Schmittgen, 2001).

**TABLE 1:** Primer sequences.

	**Primer sequence 5′→3′**		
**Primer**	**Forward**	**Reverse**	**Application**
RPS18	GCGGCGGAAAATAGCCTTTG	GATCACACGTTCCACCTCATC	mRNA analysis
RNase1	GCTGCAGATCCAGGCTTTTCTGGG	AATTTCTTGGCCCGGGATTC	mRNA analysis
COX-2	TCCCTTGGGTGTCAAAGGTAAA	TGGCCCTCGCTTATGATCTG	mRNA analysis
IL-8	ACTGAGAGTGATTGAGAGTGGAC	AACCCTCTGCACCCAGTTTTC	mRNA analysis
CSNK2A1	GAGATTCTGAAGGCCCTGGATT	ACTCAGCCAAACCCCAGTCT	mRNA analysis
CSNK2A2	CAACTATGACCAGCTTGTTCGC	TTTCCCAGCGTTTCCGTGA	mRNA analysis
CSNK2B	GCAGGTCCCTCACTATCGAC	ACTTTTCCAACATCTGGGCG	mRNA analysis
RCOR1	TCGCCGTACAAGCCATCAGG	ACCATGTTCTGCCTCCCATTC	mRNA analysis
SIN3A	GCTCCAGCTATCGAGCCTTA	ACATCAAGCTCAAAGCGTTC	mRNA analysis
SIN3B	TGCTTCAAGGTGATGTTCCTG	CATACTGCTCCACGTACCGA	mRNA analysis
CHD3	CAGCCACGGTTTATCACAGC	ACCTTTTGTGTGGCCCTCC	mRNA analysis
CHD4	CCCCGAGAGGTTCCACAAT	CTCGGGCATTGAGTGCTTCA	mRNA analysis
*Region A*	TGAGGAAGGAGTGGTGAATC	TTTCTCTGCTGCTCCTTGTG	ChIP
*Region B*	CATTAGATCGCCCTGTTG	TTTACACGACACGGGAGCCTTC	ChIP
*Region C*	CTGGCCCTAGGAATCCTGAAAC	CTGCAGTAAGGGCTTCTGATGG	ChIP

### Chromatin Immunoprecipitation

Chemicals used for ChIP were purchased from Carl Roth GmbH & Co., KG, unless otherwise stated. Confluent cells were stimulated as indicated and ChIP assay was performed as described previously (Bedenbender et al., 2019). In brief, cells were fixed for 5 min with 1 % formaldehyde (methanol-free; Polysciences, Inc., Warrington, PA, United States) at room temperature. Fixation was stopped with glycine (0.125 M) for 5 min at room temperature. Cells were washed and scraped with ice cold 1× PBS (without magnesium and calcium; HyClone^TM^, GE Healthcare, Solingen, NW, Germany) and centrifuged at 300 × *g* for 10 min at 4°C. Scraping and washing was repeated twice. Chemical lysis was performed using lysis buffer I [20 min at 4°C; 5 mM piperazine-N,N′-bis-(2 ethane sulfonic acid) pH 8, 85 mM potassium chloride, 0.5% Nonidet P40 (AppliChem GmbH, Darmstadt, HE, Germany)] and II [10 min at 4°C; 10 mM Tris–hydrochloride pH 7.5, 150 mM sodium chloride, 1% sodium deoxycholate, 0.1% sodium dodecyl sulfate, 1% Nonidet P40 (AppliChem GmbH)]. Chromatin was sheared by sonication, 20 cycles, each 30 s on/off using Bioruptor Plus (Diagenode SA, Seraing, LG, Belgium). Sepharose A (GE Healthcare) beads were blocked overnight at 4°C with 1 mg/ml bovine serum albumin (BSA) (Carl Roth GmbH & Co., KG) and 400 μg sonicated salmon sperm DNA (AppliChem GmbH). Sonicated chromatin was pre-cleared by incubation with blocked beads, and 10–20 μg pre-cleared chromatin were used for immunoprecipitation overnight at 4°C, using the following protein specific antibodies: anti-H4ac that recognizes acetylated histone 4 at lysine 5, 8, 12, and 16 as indicated by the manufacturer (06-598, Merck KGaA), anti-H3K27ac (ab4729), anti-CHD4 (ab72418), and anti-Rabbit IgG (ab171870; Abcam, Cambridge, United Kingdom) as indicated. Fresh beads were added to each IP for 2 h followed by diverse washing steps using washing buffer I-III (I: 20 mM Tris–hydrochloride pH 8.0, 150 mM sodium chloride, 2 mM Ethylenediaminetetraacetic acid (EDTA), 0.1% sodium dodecyl sulfate, 1% Triton X100; II: 20 mM Tris–hydrochloride pH 8.0, 500 mM sodium chloride, 2 mM EDTA, 0.1% sodium dodecyl sulfate, 1% Triton X100; III: 10 mM Tris–hydrochloride pH 8.0, 1% Nonidet P40, 1% sodium deoxycholate, 1 mM EDTA, 0.25 M Lithium chloride) and 1× Tris–EDTA buffer (10 mM Tris pH8.0, 1 mM EDTA). Chromatin-antibody complexes were chemically eluted (1% SDS, 0.1 M sodium hydrogen carbonate) and reversion of crosslinking was conducted over night by Proteinase K digestion (AppliChem GmbH) prior to DNA purification using the QIAquick PCR purification kit (QIAGEN GmbH) according to manufacturer’s instructions. Purified chromatin was eluted in H_2_O and analyzed by qPCR using indicated ChIP-primers (Table 1, metabion international AG) for human *RNASE1* promoter regions *Region A*, *Region B*, *Region C*. Results of ChIP experiments were normalized to the input control (depicted as % of input) and the respective CTRL samples with the specific antibody (CTRL or CTRL DMSO) were set to 1.

### Statistical Analyses

Statistical analyses were performed using GraphPad Prism Version 6.05 (GraphPad Software, La Jolla, CA, United States). Results are expressed as mean ± standard deviation (SD) of linear data. Statistical analyses of mRNA data were conducted on log2-transformed data. One-way or two-way ANOVA and subsequent multiple comparison using Sidak post-test or Holm-Sidak post-test, respectively, were performed as indicated. Results were considered significant at *p* < 0.05.

## Results

### p38 MAPK Signaling Mediates RNase1 mRNA Repression in Inflamed Human ECs

**FIGURE 1:**
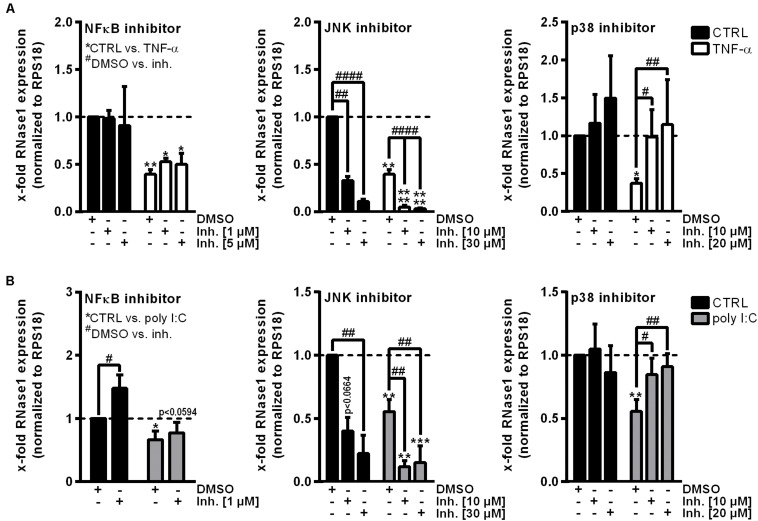
p38 inhibitor treatment restores RNase1 mRNA expression in inflamed human ECs. HUVEC were pretreated for 1 h with indicated signaling pathway inhibitors or DMSO as solvent control prior to **(A)** TNF-α [10 ng/ml] (white bars) or **(B)** poly I:C [10 μg/ml] (gray bars) stimulation for 24 h or left untreated as control (black bars). Expression of RNase1 mRNA was analyzed by qRT-PCR. Results were normalized to RPS18 and the respective CTRL samples; *n* = 3–4; mean ± standard deviation (SD); Statistics were performed on log2-transformed data. Two-way ANOVA was performed using Holm-Sidak post-test. *CTRL vs. stimulus: *p < 0.05, **p < 0.01, ***p < 0.001, ****p < 0.0001; ^#^DMSO vs. inhibitor (inh.): ^#^*p* < 0.05, ^##^*p* < 0.01, ^####^*p* < 0.0001.

To investigate the underlying molecular mechanisms of RNase1 regulation in inflamed human ECs, we first aimed to identify relevant signaling pathways for this process. Besides TNF-α or IL-1β stimulation of primary human ECs, treatment with the toll-like receptor 3 ligand poly I:C for 24 h also resulted in downregulation of RNase1 mRNA (Bedenbender et al., 2019). Here, poly I:C stimulation kinetics in HUVEC from 0.5 to 24 h resulted in a significant downregulation of RNase1 mRNA abundance after 9 h of treatment that further intensified over time until 24 h (Supplementary Figure 1A). These findings are comparable to the previously observed RNase1 regulation in TNF-α or IL-1β stimulation kinetics (Bedenbender et al., 2019). Based on these findings, common signaling cascades induced upon all three RNase1 repressive stimuli were investigated: NF-κB signaling or MAPK signaling via JNK and p38 kinases (Kawai and Akira, 2006; Weber et al., 2010; Brenner et al., 2015). HUVEC were pre-stimulated for 1 h with indicated concentrations of the signaling pathway inhibitors BAY11-7082 (NF-κB inhibitor) as negative control (Gansler et al., 2014), JNK inhibitor II (JNK MAPK inhibitor), SB202190 (p38 MAPK inhibitor) or DMSO as solvent control prior to 24 h TNF-α (white bars; Figure 1A) or poly I:C (gray bars; Figure 1B) stimulation, or left untreated as control (CTRL; black bars). In accordance with previous reports (Gansler et al., 2014), RNase1 mRNA expression was not affected by NF-κB inhibitor treatment in CTRL as well as TNF-α stimulated cells, compared to the DMSO control (Figure 1A, left panel). In respect to JNK inhibitor treatment, significantly diminished RNase1 mRNA expression was detected in CTRL cells compared to DMSO in a dose-dependent manner, while TNF-α-mediated RNase1 repression was even stronger after JNK inhibition (Figure 1A, middle panel). In contrast to these results, pretreatment of HUVEC with the p38 inhibitor slightly increased RNase1 mRNA abundance in CTRL cells compared to DMSO treatment. Moreover, TNF-α-mediated downregulation of RNase1 compared to the solvent control was completely restored dose-dependently by p38 inhibition (Figure 1A, right panel). In addition, comparable results to TNF-α treatment were obtained for RNase1 mRNA abundance by stimulation with the toll-like receptor 3 ligand poly I:C following NF-κB, JNK and p38 inhibitor stimulation compared to the solvent control DMSO as well as CTRL treated cells (Figure 1B). However, NF-κB inhibitor treatment slightly increased RNase1 expression in CTRL treated cells, compared to DMSO (Figure 1B, left panel). To validate the obtained results with the p38 inhibitor, we also investigated the expression of the p38-dependent gene COX-2, which is known to be upregulated upon proinflammatory stimulation of HUVEC (Viemann et al., 2004). Here, COX-2 was significantly upregulated upon TNF-α as well as poly I:C stimulation in DMSO treated samples, while p38 inhibition considerably blocked COX-2 induction upon proinflammatory treatment (Supplementary Figure 1B, left panel). Accordingly, physiological RNase1 expression seemed to be regulated via JNK signaling pathway, while inflammation-mediated RNase1 repression was mediated in a p38 MAPK-dependent manner in human ECs.

### Inflammation-Mediated Repression of RNase1 in Human ECs Is Conducted by CK2 Kinase

**FIGURE 2:**
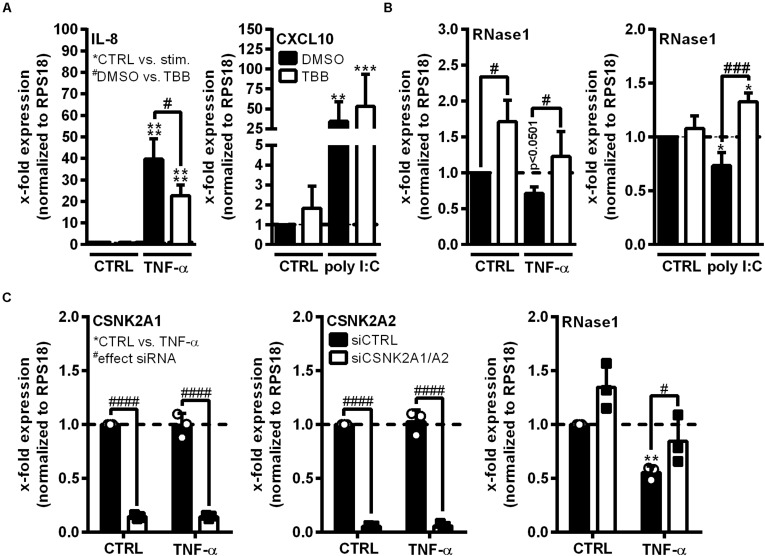
CK2 function is required for RNase1 repression in inflamed human ECs. **(A,B)** EA.hy926 were pretreated for 30 min with CK2 inhibitor TBB [10 μM] (white bars) or DMSO as solvent control (black bars), prior to TNF-α [10 ng/ml] or poly I:C [10 μg/ml] stimulation for 24 h or left untreated as control (CTRL). **(C)** EA.hy926 were transfected with siRNA pools against both CSNK2A1 and CSNK2A2 (each 25 pmol; siCSNK2A1/A2; white bars) or 50 pmol scrambled siRNA control pool (siCTRL; black bars) for 24 h followed by additional 24 h stimulation with 10 ng/ml TNF-α or left untreated as control (CTRL). mRNA expression of **(A)** IL-8 (left panel) or CXCL10 (right panel), **(B)** RNase1, **(C)** CSNK2A1 (left panel), CSNK2A2 (middle panel) or RNase1 (right panel) was analyzed by qRT-PCR. Results were normalized to endogenous RPS18 and respective CTRL samples. *n* = 3–4; mean ± SD; Statistics were performed on log2-transformed data. Two-way ANOVA was performed using Holm-Sidak post-test. *effect of TNF-α or poly I:C: *p < 0.05, **p < 0.01, ***p < 0.001 ****p < 0.0001; ^#^effect of TBB or siRNA: ^#^*p* < 0.05, ^###^*p* < 0.001, ^####^*p* < 0.0001.

Ribonuclease 1 repression upon EC inflammation is mainly attributed to the function of HDAC2, however, it is still unknown how HDAC2 activity is regulated in this context (Bedenbender et al., 2019). HDAC2 activity highly depends on its phosphorylation state and the association with multiprotein complexes, both described to be regulated by CK2 kinase (Tsai and Seto, 2002; Brandl et al., 2009). Thus, we investigated whether CK2 functions as intermediate step in the TNF-α-mediated signaling cascade to facilitate RNase1 repression. The human EC line EA.hy926 was pretreated for 30 min with 10 μM CK2 inhibitor TBB (white bars) or DMSO (black bars) as solvent control, followed by 24 h control (CTRL), TNF-α or poly I:C stimulation (Figures 2A,B). The experimental setup was validated by mRNA expression analysis of the proinflammatory marker IL-8 (for TNF-α) or CXCL10 (for poly I:C) that have been described to be partially regulated via CK2 signaling, respectively (Du et al., 2015; Li et al., 2019). IL-8 and CXCL10 mRNA were significantly upregulated in TNF-α (Figure 2A, left panel) or poly I:C (Figure 2A, right panel) treated EA.hy926 in DMSO pre-stimulated cells. Consistent with the literature, this effect was significantly reduced for IL-8 mRNA by TBB treatment upon TNF-α stimulation (Figure 2A, left panel), while CXCL10 expression upon poly I:C stimulation was further increased by TBB pretreatment (Figure 2A, right panel), both compared to the solvent control. *Vice versa*, RNase1 mRNA abundance was significantly elevated by TBB pretreatment in CTRL cells compared to the solvent control, and TNF-α-mediated reduction of RNase1 was completely recovered by TBB in contrast to DMSO (Figure 2B, left panel). Similar results were obtained for TBB and poly I:C stimulation compared to DMSO, also considerably recovering RNase1 mRNA upon inflammation (Figure 2B, right panel).

To confirm the obtained results, the influence of small interfering RNA (siRNA)-mediated CK2 knockdown on RNase1 mRNA expression was investigated. CK2 is described as an tetrameric complex of two identical or non-identical catalytic subunits (CSNK2A1/A2) and two identical regulatory subunits (CSNK2B) in humans (Litchfield, 2003). EA.hy926 were transfected for 24 h with siRNA pools against both CSNK2A1 and A2 (Figure 2B; white bars), CSNK2B (Supplementary Figures 2A,B) or an unspecific siRNA control pool (siCTRL; black bars), prior to 24 h control (CTRL) or TNF-α stimulation. Knockdown was validated by significant downregulation of respective mRNAs of CSNK2A1 (Figure 2B, left panel) and CSNK2A2 (middle panel) upon specific siRNA treatment in both control and TNF-α stimulated cells. Interestingly, double-knockdown of the catalytic subunits CSNK2A1 and A2 slightly increased RNase1 expression in unstimulated cells, compared to siCTRL. Additionally, RNase1 mRNA was significantly repressed upon TNF-α treatment in control transfected cells, while CSNK2A1/2 double-knockdown considerably recovered RNase1 expression (Figure 2B, right panel). In respect to the regulatory subunit CSNK2B, successful knockdown was validated by significant downregulation of CSNK2B mRNA (Supplementary Figure 2A) upon specific siRNA treatment in both control and TNF-α stimulated cells. In contrast to CSNK2A1/A2 double-knockdown, significant downregulation of RNase1 was obtained upon TNF-α treatment compared to CTRL, for both, siCTRL and CSNK2B transfected cells (Supplementary Figure 2B). Altogether, TBB inhibitor treatment and CSNK2A1/A2 double-knockdown indicated an important role of CK2 kinase in TNF-α-mediated RNase1 repression in inflamed human ECs.

### NuRD Co-repressor Complex Components CHD3 and 4 Are Required for RNase1 Repression in Inflamed Human ECs

**FIGURE 3:**
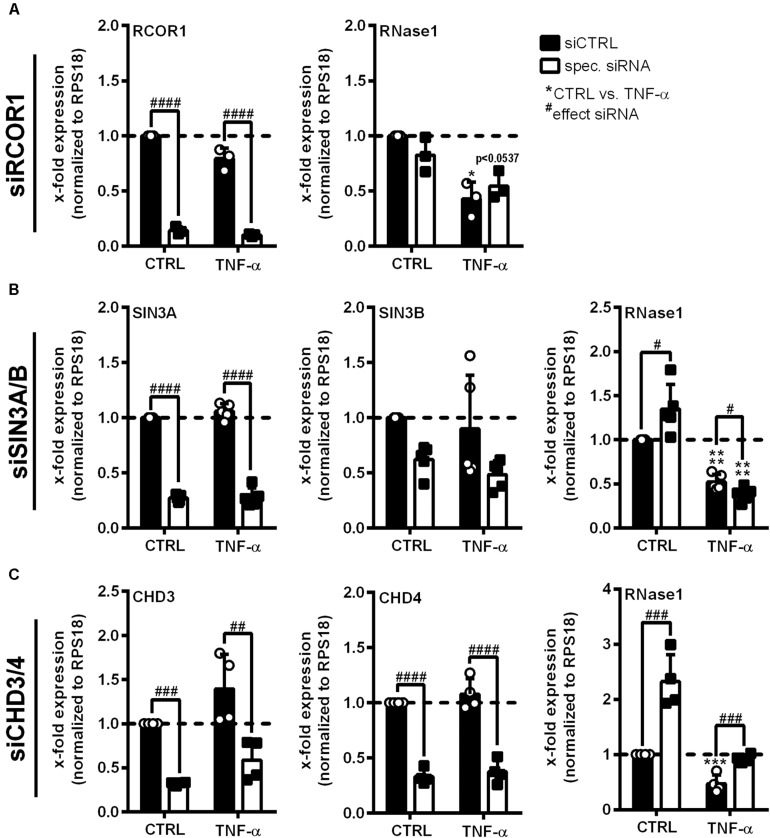
Co-repressor complex NuRD is crucial for RNase1 downregulation upon human EC inflammation. EA.hy926 were transfected with siRNA pools (50 pmol for single- or 25 pmol each for double-transfection) against **(A)** RCOR1 (siRCOR1; CoREST complex), **(B)** a combination of SIN3A and SIN3B (siSIN3A/B; SIN3 complex), **(C)** CHD3 and CHD4 (siCHD3/4; NuRD complex; white bars) or scrambled siRNA control pool (siCTRL; black bars) for 24 h followed by additional 24 h stimulation with 10 ng/ml TNF-α or left untreated as control (CTRL). mRNA expression of **(A)** RCOR1, **(B)** SIN3A and SIN3B, **(C)** CHD3 and CHD4 as well as **(A–C)** RNase1 was analyzed by qRT-PCR. Results were normalized to endogenous RPS18 and CTRL treated siCTRL samples. *n* = 3–4; mean ± SD; Statistics were performed on log2-transformed data; Two-way ANOVA was performed using Holm-Sidak post-test. *effect of TNF-α: *p < 0.05, ***p < 0.001, ****p < 0.0001; ^#^effect of siRNA: ^#^*p* < 0.05, ^##^*p* < 0.01, ^###^*p* < 0.001, ^####^*p* < 0.0001.

HDAC2 enzymatic activity requires the association with multiprotein co-repressor complexes to get access to the chromatin (Tsai and Seto, 2002; Segre and Chiocca, 2011). To identify such complexes involved in RNase1 regulation, siRNA knockdown of crucial protein components of the most abundant HDAC-associated repressor complexes was performed in EA.hy926 (Zhang et al., 1997; Tong et al., 1998; You et al., 2001; Sengupta and Seto, 2004; Yang and Seto, 2008). siRNA pools against RCOR1 (CoREST complex), SIN3A and B (double-knockdown, SIN3 complex), CHD3 and 4 (double-knockdown, NuRD complex; white bars) or an unspecific siRNA control pool (siCTRL, black bars) were transfected in EA.hy926 for 24 h, followed by additional 24 h TNF-α stimulation or left untreated as control (CTRL; Figure 3). Knockdown of distinct complex components was validated by respective mRNA analysis, resulting in significant downregulation of RCOR1 (Figure 3A, left panel), SIN3A/B (Figure 3B, left and middle panel) and CHD3/4 (Figure 3C, left and middle panel) compared to siCTRL transfection upon the tested stimuli. In respect to RNase1, no effect of RCOR1 knockdown was detected upon both treatments (Figure 3A, right panel), while SIN3A/B double-knockdown increased RNase1 mRNA levels in unstimulated cells compared to siCTRL, but rather augmented the repressive effect of TNF-α on RNase1 mRNA (Figure 3B, right panel). Remarkably, double-knockdown of the NuRD co-repressor components CHD3 and 4 significantly increased RNase1 expression in control cells as well as considerably recovered TNF-α-mediated RNase1 repression compared to siCTRL (Figure 3C, right panel). Both CHD3 and 4, alone or in combination can associate in the NuRD complex to achieve either equal or individual functions (Denslow and Wade, 2007; Hoffmeister et al., 2017). To investigate whether only one of these proteins or both are essential for the RNase1 recovering effect, we performed single-knockdown of CHD3 (Supplementary Figures 2C,D) and CHD4 (Supplementary Figures 2E,F). In respect to knockdown efficiency, comparable results were obtained by single knockdown of CHD3 or 4 as for the double-knockdown (Supplementary Figures 2C–E), as well as for RNase1 recovery (Supplementary Figures 2D–F). In conclusion, these findings suggest an essential role for both, the CHD3- and the CHD4-containing NuRD co-repressor complex in RNase1 repression upon EC inflammation, presumably promoting HDAC2 recruitment to the *RNASE1* promoter.

### CHD4 Accumulates at the RNASE1 Promoter Along With Histone Deacetylation Upon Proinflammatory Stimulation of Human ECs

**FIGURE 4:**
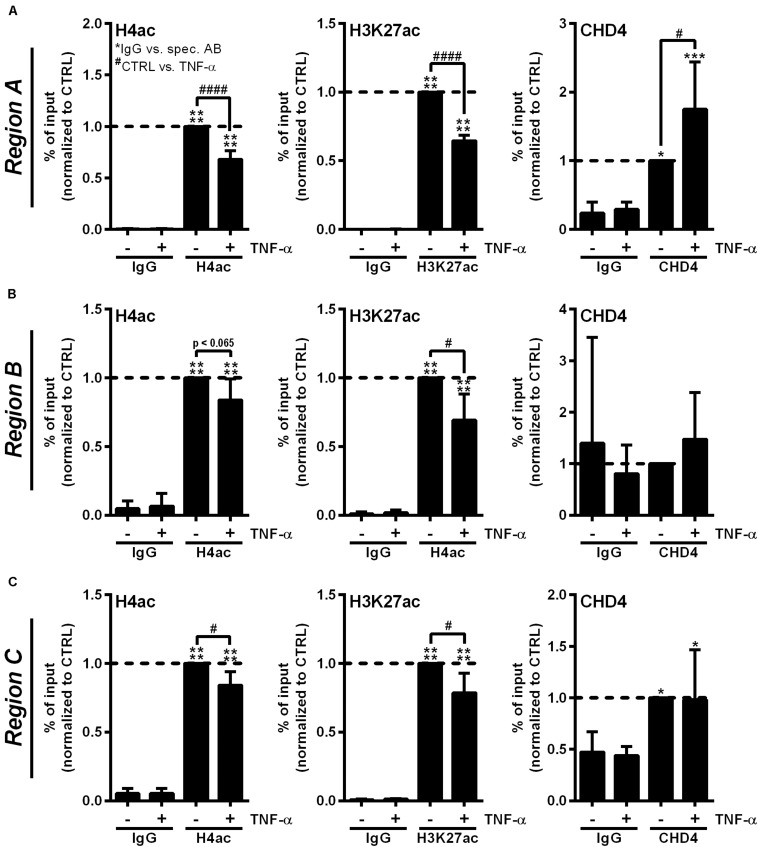
Proinflammatory stimulation of human ECs induces *RNASE1* promoter deacetylation in concert with CHD4 recruitment. HUVEC were stimulated with 10 ng/ml TNF-α for 10 min (+) or left untreated as control (–). Immunoprecipitation using specific antibodies against histone 4 acetylation (H4ac; left panels), histone 3 lysine 27 acetylation (H3K27ac; middle panels), chromodomain helicase DNA binding protein 4 (CHD4; right panels) or an unspecific IgG control was performed. **(A)** *Region A*, **(B)** *Region B*, **(C)** *Region C* of the *RNASE1* promoter were pulled down by the respective antibodies and analyzed by qPCR using respective primers. Results were depicted as % input and the respective control sample with the specific antibody was set to 1. *n* = 3–4; mean ± SD; One-way ANOVA was performed using Sidak post-test, *IgG vs. specific (spec.) antibody (AB): *p < 0.05, ***p < 0.001, ****p < 0.0001; ^#^CTRL vs. TNF-α: ^#^*p* < 0.05, ^####^*p* < 0.0001.

To confirm the participation of the CHD3/4 containing NuRD complex in RNase1 repression, we investigated *RNASE1* promoter remodeling by histone deacetylation and recruitment of NuRD complex in inflamed human ECs by analyzing the acetylation state of H4 and H3K27 as well as accumulation of the NuRD component CHD4. ChIP was performed with HUVEC stimulated for 10 min with TNF-α (+), the time of HDAC2 promoter accumulation (Bedenbender et al., 2019), or left untreated as control (−). The histone acetylation state as well as CHD4 recruitment to the previously described *RNASE1* promoter regions, *Region A* (the core promoter) and the more upstream regions *Region B* (the proximal promoter) and *Region C* (the distal promoter) (Bedenbender et al., 2019), were analyzed by qPCR. In unstimulated cells, H4 as well as H3K27 were acetylated at promoter *Region A* of *RNASE1*, while 10 min TNF-α stimulation resulted in significant deacetylation of H4 and H3K27 at the same site (Figure 4A, left and middle panel). Along with these findings, CHD4 accumulation was significantly elevated at *Region A* upon TNF-α treatment compared to untreated cells (Figure 4A, right panel). In respect to *Region B* (Figure 4B) and *C* (Figure 4C), similar results were obtained for H4ac (left panels) and H3K27ac (middle panels), while almost no or equal CHD4 accumulation was detected in control and TNF-α treated cells (Figures 4B,C, right panels). Consequently, *RNASE1* promoter *Region A–C* is markedly deacetylated after 10 min of TNF-α stimulation. This effect went along with specific recruitment of CHD4 to *Region A* of *RNASE1*, indicating a role of the CHD4 containing NuRD complex in *RNASE1* regulation in inflamed human ECs.

### Inhibition of p38 MAPK Signaling Restores Histone Acetylation at the RNASE1 Promoter and Prevents CHD4 Accumulation

**FIGURE 5:**
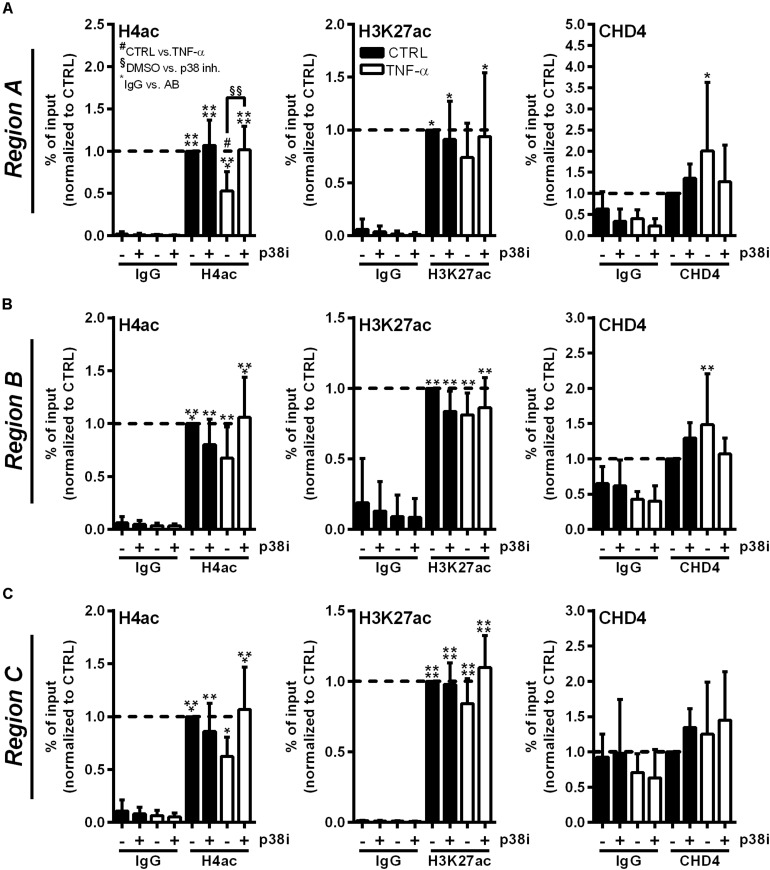
p38 inhibitor treatment reverses *RNASE1* promoter deacetylation and CHD4 recruitment in inflamed human ECs. HUVEC were pretreated with 20 μM p38 inhibitor (inh.) SB202190 (+) or DMSO as solvent control (–) for 2 h, followed by 10 min TNF-α stimulation [10 ng/ml] (white bars) or left untreated as control (CTRL; black bars). Immunoprecipitation using specific antibodies against histone 4 acetylation (H4ac; left panels), histone 3 lysine 27 acetylation (H3K27ac; middle panels), chromodomain helicase DNA binding protein 4 (CHD4; right panels) or an unspecific IgG control was performed. **(A)** *Region A*, **(B)** *Region B*, **(C)** *Region C* of the *RNASE1* promoter were pulled down by the respective antibodies and analyzed by qPCR using respective primers. Results were depicted as % input and the respective control sample with the specific antibody was set to 1. *n* = 3–4; mean ± SD; Two-way ANOVA was performed using Holm-Sidak post-test. *IgG vs. specific (spec.) antibody (AB): *p < 0.05, **p < 0.01, ***p < 0.001, ****p < 0.0001; ^#^CTRL vs. TNF-α: ^#^*p* < 0.05; ^§^DMSO vs. p38 inh.: ^§§^*p* < 0.01.

To investigate whether the described *RNASE1* promoter remodeling and CHD4 accumulation depend on p38 MAPK signaling, ChIP assays with p38 inhibition were performed. Therefore, HUVEC were pretreated for 2 h with 20 μM p38 inhibitor SB202190 (+) or DMSO (−) as solvent control, followed by 10 min stimulation with 10 ng/ml TNF-α (white bars) or left untreated as control (CTRL; black bars). Acetylation of H4 or H3K27, and the recruitment of NuRD co-repressor complex component CHD4 (right panels) were investigated by immunoprecipitation and analyzed by qPCR using respective promoter primers. Compared to inhibitor and DMSO treatment in CTRL cells, TNF-α stimulation resulted in significant deacetylation of H4 in DMSO treated samples at *Region A* of the *RNASE1* promoter. This effect was considerably restored by p38 inhibition upon TNF-α stimulation, resulting in comparable H4 acetylation as in untreated cells (Figure 5A, left panel). Similar effects were obtained for H4ac in *Region B* and *C* of the *RNASE1* promoter, although not significantly (Figures 5B,C, left panel). With respect to H3K27, reduced acetylation at *Region A–C* was observed after proinflammatory stimulation of HUVEC compared to DMSO treated CTRL cells. Although no significant recovery of H3K27ac by p38 inhibition was detected after 10 min TNF-α stimulation, an increasing trend toward restoration of acetylation was observed in *Region A–C* (Figures 5A-C, middle panel). In context of CHD4, TNF-α treatment tented to increase recruitment to the *RNASE1* promoter *Region A* compared to the DMSO treated CTRL, while p38 inhibition inclined to reverse this effect to only basal CHD4 levels (Figure 5A, right panel). At the upstream promoter *Region B*, a similar trend can be observed for CHD4 upon TNF-α stimulation, indicated by an increase in CHD4 accumulation in solvent control treated cells and its prevention upon p38 inhibition (Figure 5B, right panel). Compared to that, CHD4 was almost absent from *Region C* upon all indicated treatments (Figure 5C, right panel). These findings provide evidence that proinflammatory stimulation of HUVEC with TNF-α induced deacetylation of H4 and H3K27 at the *RNASE1* promoter as well as recruitment of the NuRD/CHD4 co-repressor complex. Interestingly, these effects can be reversed by p38 inhibition, underlining the importance of p38 signaling in *RNASE1* promoter remodeling and repression.

## Discussion

Cardiovascular disease, such as atherosclerosis, thrombosis, or myocardial infarction, that are associated with endothelial dysfunction represent a global health issue, causing approximately 18 million deaths per year worldwide^1^. Thereby, disease development and progression tightly associate to the loss of vascular function, homeostasis, and integrity in consequence to inflammation, infection, or injury of the endothelium (Poredos, 2002; Sitia et al., 2010; Zernecke and Preissner, 2016). Thus, investigation of the underlying mechanisms of endothelial dysfunction upon vascular inflammation is an important need. In this study, we investigated the underlying mechanisms of repression of vasoprotective RNase1 in context of vascular inflammation (Gansler et al., 2014; Zernecke and Preissner, 2016). We identified a regulatory mechanism by which proinflammatory stimulation, such as TNF-α or poly I:C, activated p38 MAPK, CK2 kinase, and subsequent recruitment of the HDAC2 containing NuRD/CHD4 co-repressor complex to the *RNASE1* promoter. This resulted in chromatin remodeling via histone deacetylation and subsequent RNase1 repression. In recent years, the RNase1-eRNA system was identified as crucial factor in diverse pathologies, ranging from cardiovascular diseases, such as thrombosis, atherosclerosis or myocardial infarction, to inflammatory and infectious disorders like sepsis or bacterial infections (Kannemeier et al., 2007; Cabrera-Fuentes et al., 2014; Simsekyilmaz et al., 2014; Zakrzewicz et al., 2016; Zernecke and Preissner, 2016; Zechendorf et al., 2020). In this context, RNase1 has been demonstrated as potent regulator and protective factor of vascular homeostasis by counteracting the danger-associated molecule eRNA to compensate changes in the eRNA-RNase1 system (Zernecke and Preissner, 2016). Thereby, RNase1 protects the endothelium from overwhelming inflammation by e.g., decreasing myocardial infarction size or thrombus formation, mainly via reduction of circulating eRNA levels, inflammatory cells and cytokines to recover physiological organ functions (Fischer et al., 2007; Kannemeier et al., 2007; Cabrera-Fuentes et al., 2014, 2015; Chen et al., 2014). However, RNase1 function is significantly impaired upon long-term vascular inflammation due to increased extracellular eRNA levels that further result in loss of vascular integrity (Gansler et al., 2014). These processes were shown to be mainly regulated by an HDAC-dependent mechanism by which HDAC2 is recruited to the *RNASE1* promoter region to conduct histone deacetylation, resulting in a condensed chromatin structure, loss of Polymerase II transcription machinery binding and subsequent gene repression (Bedenbender et al., 2019). Therefore, unraveling the underlying molecular mechanisms of RNase1 repression in inflamed human ECs is of great importance, to offer new therapeutic options and treatment strategies to preserve RNase1 function upon vascular inflammation.

In this study, we identified the well-known MAPK phosphorylation cascades via JNK and p38 as potent regulators of RNase1 expression in human ECs under physiological as well as proinflammatory conditions. MAPK signaling via JNK and p38 is directly associated to the RNase1 repressive proinflammatory stimuli TNF-α, IL-1β, and poly I:C (Ashwell, 2006; Kawai and Akira, 2006; Weber et al., 2010; Brenner et al., 2015; Bedenbender et al., 2019). As blocking of JNK signaling strongly repressed RNase1 expression in untreated ECs, this signaling cascade seems to be essential for physiological RNase1 expression. These findings are supported by publications, demonstrating JNK as potent regulator of EC gene expression not only upon inflammation, for instance during thrombin induced intercellular adhesion molecule 1 expression (Miho et al., 2005), but also under physiological conditions, e.g., in context of EC motility (Shin et al., 2001). However, our findings indicate that JNK inhibition upon TNF-α and poly I:C treatment potentiates the negative effect of these stimuli on RNase1 mRNA. Thus, it can be speculated that the JNK cascade might still conduct residual RNase1 mRNA expression even upon inflammation. In addition, our results confirmed an p38-dependent regulatory mechanism for RNase1 repression upon inflammation of human ECs, as demonstrated by clear recovery of RNase1 expression after TNF-α and poly I:C treatment by p38 inhibition. These findings are comparable to previous results using the HDAC1-3 inhibitor MS275 that also fully recovered RNase1 expression upon inflammation (Bedenbender et al., 2019). p38 MAPK is generally associated to inflammatory events, as it was firstly described in context of proinflammatory cytokine synthesis in endotoxin stimulated macrophages (Lee et al., 1994). Besides its crucial role in induction of proinflammatory mediators, e.g., reduced IL-6 production in p38α-deficient embryonic stem cells, or p38-dependent COX-2 expression in TNF-α stimulated HUVEC (Allen et al., 2000; Viemann et al., 2004), also repressive functions of p38 signaling, such as the reduction of certain myogenic genes during muscle differentiation in myeloid cells, are described (Suelves et al., 2004). These findings support our results that inflammation-mediated RNase1 repression is primarily associated to p38 MAPK-dependent signaling in human ECs.

To unravel further intermediated steps in the RNase1 repression cascade upon EC inflammation, we analyzed the regulation of HDAC2 activity in this context. Previous findings by our group indicated that HDAC2 activity might be regulated independent of HDAC2 mRNA or protein levels (Bedenbender et al., 2019 and unpublished data), suggesting an alternative regulatory mechanism. Class I HDAC activity, including HDAC2, can be regulated through subcellular localization, association in protein complexes and post-translational modifications. Since HDAC2 is mainly located in the nucleus due to its nuclear localization sequence and the missing export sequence (Micelli and Rastelli, 2015), we focused on its post-translational modification and association into multiprotein co-repressor complexes. Both processes can be regulated via phosphorylation of HDAC2 C-terminal serine residues by the highly conserved and constitutively active protein kinase CK2 (Tsai and Seto, 2002; Litchfield, 2003; Segre and Chiocca, 2011). CK2 function is directly associated with TNF-α and p38 signaling to mediate inflammatory processes as demonstrated in context of stress-induced human cervical carcinoma cells and diabetic retinopathy in retinal ECs (Sayed et al., 2000; Litchfield, 2003; Meggio and Pinna, 2003; Zhang and Steinle, 2014). Here, we investigated the impact of CK2 on RNase1 expression by CK2 inhibition and siRNA knockdown in the human EC line EA.hy926. Blocking of CK2 function by the inhibitor TBB as well as knockdown of the two catalytic subunits CSNK2A1/A2 significantly recovered RNase1 mRNA abundance upon TNF-α (or poly I:C) treatment, demonstrating an important role of CK2 kinase in the inflammation-mediated repression of RNase1. These findings are further supported by previously described functions of CK2 in context of inflammation, e.g., regulation of leukocyte interactive proteins, like endothelial selectin upon TNF-α-mediated EC activation or HDAC2 phosphorylation in hypoxia-associated tumors (Pluemsampant et al., 2008; Ampofo et al., 2015). To further test for a direct impact of CK2 on HDAC2 phosphorylation in inflamed ECs we performed Western Blot analysis upon CK2 inhibitor (TBB) treatment and proinflammatory stimulation of human ECs (data not shown). Although we found high HDAC2 phosphorylation levels in general, we did not observe any significant regulation. This suggests that HDAC2 activity might not be regulated exclusively by CK2-mediated phosphorylation but rather includes additional mechanisms like assembly of co-repressor complexes or cross-talk between post-translational modifications, as demonstrated for acetylation or ubiquitination. Within such complexes, HDAC2 activity has been shown to be influenced by formation of HDAC1:HDAC2 heterodimers, in which acetylated HDAC1 can control HDAC2 activity, or by combinations of HDAC2 modifications like phospho-acetylation (Adenuga and Rahman, 2010; Segre and Chiocca, 2011). In respect to a potential HDAC1:2 heterodimer formation and a possible functional redundancy of these enzymes, we already analyzed the impact of HDAC1 on RNase1 regulation in our previous publication (Bedenbender et al., 2019). Although, only HDAC2 accumulated at the *RNASE1* promoter, only siRNA double knockdown of both HDAC1 and HDAC2 recovers RNase1 mRNA expression. These findings implicate that HDAC2 is the most relevant enzyme in inflammation-mediated RNase1 regulation, however, HDAC1 might take over its function redundantly, once HDAC2 is missing (Bedenbender et al., 2019). Based on the literature, both enzymes, HDAC1 and HDAC2, can be regulated by CK2, while HDAC1 can also be regulated by other kinases (e.g., cAMP-dependent protein kinase; Tsai and Seto, 2002; Khan et al., 2013). In conclusion, our findings provide evidence that CK2 kinase is involved in regulation of RNase1 upon EC inflammation, presumably via regulation of HDAC2 activity. The interaction between CK2 and HDACs for *RNASE1* promoter remodeling remains to be clarified in detail.

As mentioned before, HDAC2 function and recruitment to promoter sites are associated with the formation of multiprotein co-repressor complexes, which in turn might also co-localize with CK2 (Grozinger and Schreiber, 2002; De Ruijter et al., 2003; Sengupta and Seto, 2004; Sun et al., 2007). Therefore, we investigated the impact of the three major HDAC2-associated repressor complexes on RNase1 mRNA expression upon EC inflammation via siRNA-mediated knockdown of essential co-repressor complex components: RCOR1 for CoREST, SIN3A/B for SIN3 and CHD3/4 (also known as Mi2α/β) for NuRD (Zhang et al., 1997; Tong et al., 1998; Humphrey et al., 2001; You et al., 2001). Only single- or double-knockdown of the NuRD repressor complex components CHD3 and 4 significantly restored RNase1 mRNA abundance upon TNF-α treatment in our experimental setting. Hence, our findings imply an important involvement of NuRD-associated CHD3 and CHD4 in RNase1 repression in inflamed human ECs. These findings are supported by the essential roles of NuRD in context of inflammation, e.g., in regulation of T- and B-cell development or macrophage immune responses (Fujita et al., 2004; Shimizu-Hirota et al., 2012; Shen et al., 2018). The great impact of NuRD co-repressor complex in RNase1 regulation is further confirmed by recruitment of CHD4 to the *RNASE1* promoter *Region A* upon 10 min TNF-α treatment. Additionally, this recruitment goes along with deacetylation of H4 and H3K27, two well-known markers of actively transcribed chromatin, that are already associated to regulation of *RNASE1* (Kouzarides, 2007; Wang et al., 2008; Bedenbender et al., 2019). Among the diverse types of co-repressor complexes, the NuRD chromatin-remodeling complex is unique due to the combination of chromatin-remodeling enzymes, like the DNA helicase-like ATPases CHD3 and CHD4, and histone modifying subunits, such as HDAC2 (Tong et al., 1998; Denslow and Wade, 2007). Hence, the obtained results for CHD4 are in accordance with the previously described accumulation of HDAC2 at the *RNASE1* promoter *Region A* after 10 min of TNF-α treatment (Bedenbender et al., 2019). Although HDAC2 also accumulated at *Region B* of the *RNASE1* promoter in previous experiments (Bedenbender et al., 2019), CHD4 seemed to be absent in this region under the same conditions. In this context, however, we are aware that ChIP analysis using primary ECs can be highly susceptible to donor variances of self-isolated primary cells. These effects might yield in high standard deviations and therewith potentially masking small differences in CHD4 recruitment to this region. Despite these small discrepancies, our current data indicate that HDAC2 recruitment to the *RNASE1* promoter is mainly mediated via the CHD3/4 containing NuRD complex upon inflammation of human ECs. To further strengthen these findings, the impact of NuRD knockdown or inhibition on HDAC2 recruitment and subsequent chromatin remodeling at the *RNASE1* promoter should be considered for future investigations. Although there are only few publications regarding NuRD functions in vascular ECs, the impact of this complex in context of vascular integrity seems to be crucial. Ingram et al. (2013) demonstrated a preventive function of NuRD/CHD4 on excessive extracellular matrix proteolysis, while Colijn et al. (2020) observed protection of vascular integrity by NuRD/CHD4-mediated histone deacetylation and gene repression upon hypoxia in embryonic ECs. Altogether, the presented data and previous findings of our group suggest that simultaneous recruitment of HDAC2 and CHD4 within the NuRD complex induces chromatin remodeling like deacetylation of H4 and H3K27 that finally result in a condensed chromatin structure, loss of polymerase transcription machinery binding and subsequent *RNASE1* repression in inflamed human ECs (Bedenbender et al., 2019).

Since p38 inhibition in HUVEC illustrated the involvement of this signaling cascade in inflammation-mediated RNase1 repression, we wondered whether p38 inhibition was also able to reverse *RNASE1* chromatin remodeling with respect to histone acetylation and CHD4 recruitment. Using ChIP analysis in primary human ECs, we found that H4ac was restored by p38 inhibitor treatment at the *RNASE1* promoter despite TNF-α stimulation. With respect to H3K27ac, a trend toward recovery of acetylation was also induced upon p38 inhibition. However, the weak effect on H3K27ac could be due to kinetics, as H4 acetylation might be recovered prior to H3K27ac. These findings are in line with previous studies from our group that demonstrated that inhibition of HDAC1, HDAC2 and HDAC3 with the class I HDAC inhibitor MS275 significantly recovered histone acetylation after 30 min of TNF-α treatment (Bedenbender et al., 2019). Comparing the current findings with our previous work, stronger effects in respect to histone acetylation upon p38 inhibitor treatment might be obtained after 30 min of TNF-α stimulation. In general, these findings illustrate the role of p38 inhibition for the recovery of histone acetylation at the *RNASE1* promoter, especially at H4, and presumably also at H3K27. Additionally, p38 inhibition reduced inflammation-induced accumulation of CHD4 at *RNASE1* promoter *Region A* and *Region B*. Compared to the presented data from Figure 4, these findings also suggest accumulation of CHD4 at the upstream promoter *Region B* which would correspond to the recruitment of HDAC2 at the same site (Bedenbender et al., 2019). Thus, CHD4 as part of the NuRD co-repressor complex was recruited to the *RNASE1* promoter in a p38-dependent manner. MAPK signaling cascades are known to be highly involved in chromatin remodeling and subsequent gene regulation, for instance via phosphorylation of transcription factors that further influence recruitment of the polymerase II transcription machinery, histone acetyl transferase complexes, as well as chromatin remodeling and HDAC complexes (Suganuma and Workman, 2011). In this context, TNF-α-induced p38 signaling was shown to promote the interaction of transcription factor YY1 and the polycomb repressive complex 2 to induce repressive chromatin structures at target gene promoters (Palacios et al., 2010), while tumor suppressor SALL1 induces p38-dependent NuRD recruitment to promote cancer cell senescence (Ma et al., 2018). Although transcription factors involved in RNase1 repression are still unknown, these findings are in line with the presented observations that TNF-α-induced p38 signaling is critical for recruitment of the NuRD/CHD4 complex to the *RNASE1* promoter to conduct H4 and H3K27 deacetylation and *RNASE1* repression.

Finally, with respect to RNase1 recovery by inhibition of CK2 and p38, our study may pave the way to new strategies to maintain vascular homeostasis in context of vascular pathologies by preserving the protective factor RNase1. Previous studies already indicated an essential role of both CK and p38 in context of vascular diseases that can be also related to studies addressing RNase1 function in the same context. For instance, both CK2 and p38 kinase have been implicated in the regulation of foam cell formation during atherosclerosis, which can be blocked by either CK2 or p38 inhibition (Zhao et al., 2002; Harvey et al., 2007). Interestingly, the macrophage content in atherosclerotic lesions can be also associated to accumulating eRNA, which can be blocked by RNase1 administration (Simsekyilmaz et al., 2014). Additionally, p38 inhibition also protects cardiomyocytes from cellular injury upon myocardial ischemia and reperfusion, or reduces excessive inflammatory cytokine expression (e.g., TNF-α) and infarct size during stroke (Barone et al., 2001; Kaiser et al., 2004, 2005). Remarkably, RNase1 administration reduces myocardial as well as cerebral infarction size and preserves cellular function in the same pathological context (Walberer et al., 2009; Cabrera-Fuentes et al., 2014, 2015). Based on these findings, a close association between p38 signaling, CK2, TNF-α, eRNA and reduced RNase1 levels is likely in diverse vascular pathologies. In this context CK2, as well as p38 inhibitors are already used in clinical trials for treatment of hematological and solid cancers or chronic inflammatory diseases, like rheumatoid arthritis or diabetes mellitus, respectively (Kumar et al., 2003; Lee and Dominguez, 2005; Buontempo et al., 2018), indicating their use as promising targets also for treatment of vascular diseases.

In conclusion, we found evidence for a regulatory mechanism of RNase1 repression in inflamed human ECs by which proinflammatory stimulation induces p38 signaling to activate CK2 kinase which further promotes HDAC2 activation and association into the NuRD co-repressor complex. Consequently, NuRD/CHD4 as well as HDAC2 are recruited to the *RNASE1* promoter to facilitate chromatin remodeling via histone deacetylation, followed by chromatin condensation and transcriptional repression. Hence, identification of associated molecules in inflammation-mediated repression of the vessel-protective factor RNase1 may provide new potential targets, such as p38 MAPK, CK2 kinase, or the NuRD/CHD4 repressor complex, for treatment of cardiovascular pathologies.

## Data Availability Statement

The raw data supporting the conclusions of this article will be made available by the authors, without undue reservation.

## Ethics Statement

The studies involving human participants were reviewed and approved by the local ethics regulations of the Philipps-University Marburg (permit number: AZ 20/16). The patients/participants provided their written informed consent to participate in this study.

## Author Contributions

KB, EV, and BS contributed to conception and design of the study. KB and IB performed the research and analyzed the data. KB wrote the manuscript. IB, EV, and BS contributed to manuscript revision, read and approved the submitted version. All authors contributed to the article and approved the submitted version.

## Conflict of Interest

The authors declare that the research was conducted in the absence of any commercial or financial relationships that could be construed as a potential conflict of interest.

## Footnotes

1 https://www.who.int/news-room/fact-sheets/detail/cardiovascular-diseases-(cvds)

## Abbreviations

Ac: acetylation
CK2: casein kinase 2
CHD: chromodomain helicase DNA binding protein
ChIP: chromatin immunoprecipitation
CoREST: REST co-repressor
COX-2: cyclo-oxygenase 2
CSNK2: casein kinase 2 subunit
CTRL: control
EC: endothelial cell
eRNA: extracellular RNA
H3K27: histone 3 lysine 27
H4: histone 4
HDAC: histone deacetylase
HUVEC: human umbilical vein endothelial cells
IL: interleukin
JNK: c-JunN-terminal kinase
MAPK: mitogen-activated protein kinase
NF- κ B: Nuclear factor kappa B
NuRD: nucleosome remodeling and deacetylase
poly I:C: polyinosinic polycytidylic acid
qPCR: quantitative PCR
qRT-PCR: quantitative reverse transcription PCR
RCOR1: REST co-repressor 1
RNase1: ribonuclease 1
SIN3: SIN3 transcription regulator family member
siRNA: small interfering RNA
TNF- α: tumor necrosis factor alpha.
