# Supplementary material for: p38 and Casein Kinase 2 Mediate Ribonuclease 1 Repression in Inflamed Human Endothelial Cells via Promoter Remodeling Through Nucleosome Remodeling and Deacetylase Complex
Source: Front Cell Dev Biol. 2020 Oct 15;8:563604. doi: 10.3389/fcell.2020.563604 (PMC7593526; doi:10.3389/fcell.2020.563604)
Supplement: Supplementary file 1 [file Table_1.docx]

Supplementary Material





**Supplementary Figure 1: Regulation of RNase1 and COX-2 mRNA expression in inflamed human ECs.** **(A)** HUVEC were stimulated with poly I:C [10 µg/ml] for 0.5, 1, 3, 6, 9, or 24 h or left untreated as control. **(B)** HUVEC were pretreated with indicated concentrations of p38 inhibitor or DMSO as solvent control for 1 h prior to TNF-α [10 ng/ml] (white bars) or poly I:C [10 µg/ml] (gray bars) stimulation for 24 h or left untreated as control (CTRL, black bars). Expression of **(A)** RNase1 or **(B)** COX-2 mRNA was analyzed by qRT-PCR. Results were normalized to RPS18 and the respective CTRL samples. n = 3-4; mean ± standard deviation (SD); Statistics were performed on log2‑transformed data. Two‑way ANOVA was performed using Holm‑Sidak posttest. **(A)** Significance for poly I:C: ^*^p < 0.05, ^**^p < 0.001. **(B)** ^*^CTRL vs. stimulus: ^*^p < 0.05, ^**^p < 0.01, ^****^p < 0.0001; ^#^DMSO vs. inhibitor (inh.): ^##^p < 0.01, ^####^p < 0.0001.





**Supplementary Figure 2: Impact of CSNK2B, CHD3 and CHD4 siRNA knockdown on RNase1 mRNA expression in inflamed human ECs.** EA.hy926 were transfected with 50 pmol siRNA pools against **(A‑B)** CSNK2B (CK2 subunit B), **(C‑D)** CHD3 or **(E‑F)** CHD4 (NuRD complex component; white bars) or scrambled siRNA control pool (siCTRL; black bars) for 24 h followed by additional 24 h stimulation with 10 ng/ml TNF‑α or left untreated as control (CTRL). mRNA expression of **(A)** CSNK2B, **(C)** CHD3, **(E)** CHD4 (upper panels) as well as **(B, D, F)** RNase1 (lower panels) was analyzed by qRT-PCR. n = 3; mean ± SD; Results were normalized to endogenous RPS18 and CTRL treated siCTRL samples. Statistics were performed on log2-transformed data; Two-way ANOVA was performed using Holm‑Sidak posttest. ^*^CTRL vs. TNF‑α: ^*^p < 0.05, ^**^p < 0.01, ^***^p < 0.001, ^****^p < 0.0001; ^#^effect of siRNA: ^###^p < 0.001, ^####^p < 0.0001.


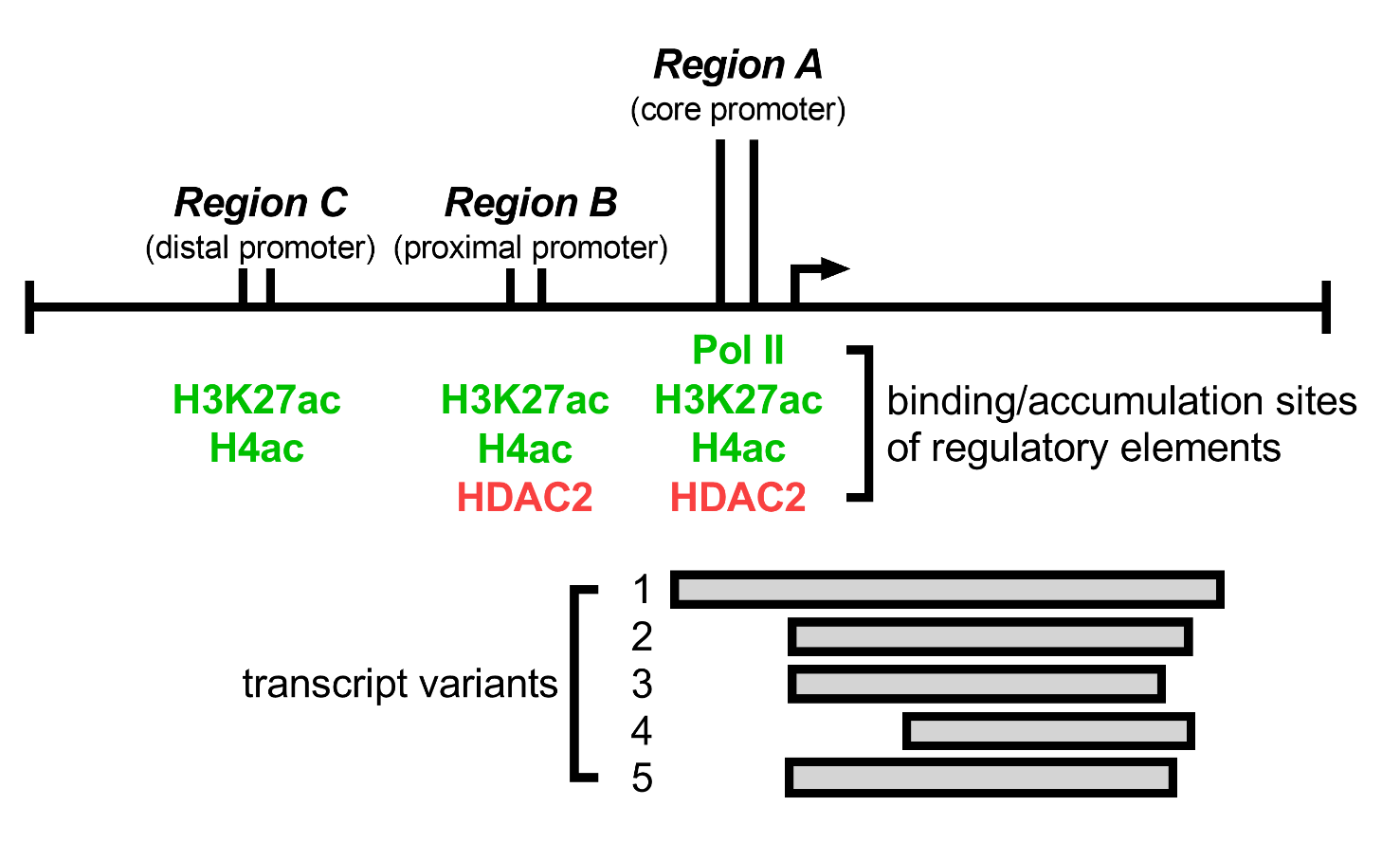


**Supplementary Figure 3: Schematic illustration of the *RNASE1* gene region.** Simplified scheme of the *RNASE1* genomic region and transcript variants (1-5) based on the UCSC Genome Browser (Human Feb. 2009 (GRCh37/hg19) Assembly). Regulatory elements involved in *RNASE1* regulation are depicted based on Bedenbender *et al.*, 2019: H3K27ac site, H4ac site, Pol II binding site (inducing marks depicted in green), HDAC2 accumulation site (repressive mark depicted in red). Primer pairs amplifying the different *RNASE1* promoter regions, named *Region A* (core promoter), *Region B* (proximal promoter) and *Region C* (distal promoter) were used for ChIP analysis (Bedenbender *et al.*, 2019). H3K27ac: histone 3 lysine 27 acetylation; H4ac: histone 4 acetylation; Pol II: RNA polymerase II.
